# Dr. C. B. Michener

**Published:** 1891-07

**Authors:** 


### EDITORIAL DEPARTMENT.

### DR. C. B. MICHENER.

We congratulate the Bureau of Animal Industry and Dr. C. B. Michener, on their mutual choice and acceptance, in the latter's appointment, as Assistant Chief of the Bureau of Animal Industry.

Dr. Michener has been well known as a New York practitioner, as Professor of the American Veterinary College, as Professor of the New York College of Veterinary Surgeons, and as an employee of the Bureau of Animal Industry, stationed in New York. His departure from New York will be a personal loss to his many friends, but his assuming charge of the business portion of the Bureau of Animal Industry, will be a gain to the government and, we trust, agreeable to himself.

